# Visual Search Performance in Children With ASD: A Combined Case‐Control and Longitudinal Study

**DOI:** 10.1002/aur.70274

**Published:** 2026-05-12

**Authors:** Naisan Raji, Iskra Todorova, Leonie Polzer, Solvejg K. Kleber, Christian Lemler, Luisa Schnettler, Janina Kitzerow‐Cleven, Ziyon Kim, Christine M. Freitag, Nico Bast

**Affiliations:** ^1^ Department of Child and Adolescent Psychiatry, Psychosomatics and Psychotherapy University Hospital Frankfurt, Goethe‐University Frankfurt Frankfurt am Main Germany; ^2^ Department of Child and Adolescent Psychiatry, Psychosomatics and Psychotherapy University Hospital Wuerzburg Wuerzburg Germany

**Keywords:** attention, autism spectrum disorder, eye‐tracking, social communication, visual perception

## Abstract

Autism Spectrum Disorder (ASD) is associated with altered attentional function. This has been associated with enhanced visual search performance. Addressing a gap in the literature, we investigate the longitudinal development of visual search alongside ASD symptoms. Using eye‐tracking, we studied performance in a single‐feature visual search task with visual search accuracy and time to target. We investigated preschoolers with ASD (*n* = 60, age = 47 months) and a cohort of developmental age‐matched typically developing (TD) children (*n* = 50, age = 35 months) at baseline and after approximately 3 years. We further explored associations of visual search performance with parent‐reported ASD symptom domains. Both groups showed similar visual search accuracy and time to target at baseline. At the 3‐year follow‐up, the ASD group showed significantly lower visual search accuracy and shorter time to target than the TD group. Within the ASD cohort, higher accuracy and shorter time to target were associated with higher social communication impairments. The often‐reported ASD advantage in visual search may not be as pronounced as assumed to differentiate between ASD and neurotypical development. It may also be dependent on the measure reported. More accurate and faster search may be associated with certain symptom severity profiles of ASD, supporting broad heterogeneity in the autism spectrum.

## Summary

Children with autism spectrum disorder (ASD) have been reported to show better performance in visual search tasks than typically developing (TD) children without ASD. Using eye‐tracking at two time points over 3 years, we found a more complex pattern. While children with ASD and TD children show similar performance at preschool age, children with ASD show lower visual search accuracy but faster time to target after 3 years. Also, our findings support an association of enhanced visual search and ASD symptomatology within neurodiversity.

## 1 Introduction

Altered attentional function has been proposed to be involved in the development of reduced social communication and restricted and repetitive behavior in Autism Spectrum Disorder (ASD) (Blaser et al. 2014; Keehn, Müller, and Townsend 2013; Sacrey et al. 2014). Visual attention enables us to focus on some aspects of our environment while neglecting others (Carrasco 2011). Emerging during the first months of life (Pyykkö et al. 2019), it plays a crucial role in early development (Hendry et al. 2019). Visual attention has been shown to be altered in individuals with ASD: Faster or more accurate visual attention was observed in studies operationalizing visual search (Kaldy et al. 2011; O'Riordan et al. 2001; Plaisted et al. 1998).

Visual search requires participants to identify a visual target within a set of distractors. It has been discussed which attention functions may be involved in visual search. While the distinction between a preattentive stage of perception of basic features, and an attentive stage of directing attention for object recognition has been proposed (Treisman and Gelade 1980), it has been argued that attentive processes are present in early stages of visual processing (Wolfe and Horowitz 2004). Superior visual search in ASD has been discussed as the result of enhanced perception and discrimination (Edmondson et al. 2020; Joseph et al. 2009; O'Riordan and Plaisted 2001). With increasing search complexity, more elaborated attentive processes such as executive functions are involved by implementation of search strategies (Evans et al. 2011).

Not all studies have been able to replicate findings of superior visual search in ASD (Keehn, Shih, et al. 2013) and meta‐analyses have suggested that effects may be small (Muth et al. 2014; Van der Hallen et al. 2015). The objective assessment of visual attention via eye tracking is independent of verbal instruction or motor abilities allowing the investigation of children with a broad range of cognitive abilities and developmental stages, overcoming potential short‐comings of earlier studies. Using eye tracking, superior visual search performance was found in toddlers with ASD compared to age‐matched typically developing (TD) children (Kaldy et al. 2011).

The relevance of early visual search performance for later development of ASD symptoms has been investigated by cross‐sectional studies reporting an association of faster visual search and social communication impairments in children and adolescents with ASD (Joseph et al. 2009; Keehn, Shih, et al. 2013). Few longitudinal studies are available. In high‐risk infants, higher accuracy of visual search at 9 months was positively related to ASD symptoms at 15 months and at 2 years (Gliga et al. 2015). At follow‐up, the high‐risk infants who went on receiving a clinical ASD diagnosis at 3 years also showed higher visual search accuracy at 9 and 15 months (Cheung et al. 2018). However, the predictive value of enhanced visual search on ASD symptoms by enhanced visual search has not been shown consistently (Edmondson et al. 2020), or only in children and adolescents with average or high fine and gross motor function (Lindor et al. 2018), suggesting that enhanced visual search may not be found across the autism spectrum.

Contrasting findings on visual search may have been induced by variation in task designs, stimulus characteristics, and study designs. Studies have predominantly reported either reaction time or accuracy, mostly of manual visuo‐motor response (Kaldy et al. 2016), which has been shown to be slower in ASD (Sachse et al. 2013; Zapparrata et al. 2023) due to attenuated fine and gross motor skills (Freitag et al. 2007). Rarely, studies have reported both accuracy and reaction time (e.g., Edmondson et al. 2020). Thus, in this study, we want to explore visual search performance using a basic single‐feature visual search task via automated eye‐tracking assessment, which is not confounded by manual visuo‐motor reaction. Complementing previous studies, we assess both accuracy of visual search and the saccadic time to the target.

Previous studies investigating visual search in ASD via eye‐tracking assessment have focused on infants and toddlers (Cheung et al. 2018; Gliga et al. 2015; Kaldy et al. 2011) and school‐aged children (Lindor et al. 2018), but we know little about the development of visual search performance from toddlerhood to school age. Given the heterogeneity of ASD and its trajectories during childhood (Franchini et al. 2023; Raji et al. 2025), associated attentional function may also vary. Thus, we aim to examine how visual search performance develops in toddlers and preschool children diagnosed with ASD compared to TD controls over the course of 3 years. Based on previous findings, we expect that children with an ASD diagnosis outperform TD peers in visual search. We hypothesize higher accuracy and shorter time to target in ASD compared to TD. Additionally, we explore the stability of group differences over 3 years.

Given the mixed findings on associations between visual search performance with ASD symptom domains, we further explore the association of visual search performance and dimensional measures of ASD symptoms. Following previous studies which suggest an etiological link between visual attention alterations and ASD, we hypothesize that higher accuracy and shorter time to target will be related to increased caregiver‐reported social communication impairments and restricted and repetitive behaviors. Finally, we explore the development of these associations over time.

## 2 Methods

### 2.1 Sample

A total of *n* = 134 children (*n*_ASD_ = 68; *n*_TD_ = 66) with a mean age of 53 months (SD = 23) were assessed. Participants with ASD were ascertained for a randomized controlled trial (Freitag et al. 2025; Kitzerow et al. 2020). Typically developing children (TD), matched for nonverbal developmental age, were additionally recruited from the community (Polzer et al. 2022). The data were collected during two visits (baseline, follow up = +36 months; Table 1). In children with ASD, diagnoses according to DSM‐5 were confirmed by trained clinical psychologists by the Autism Diagnostic Interview‐Revised (Bölte et al. 2006; Rutter et al. 2003) and the Autism Diagnostic Observation Scale‐2 (Lord et al. 2012; Poustka et al. 2015). TD participants showed parent‐reported scores below the cut‐offs in the Child Behavior Checklist 1.5‐5 (*T* < 65; Achenbach 2013), or the Social Responsiveness Scale (*T* < 75; Constantino and Gruber 2012). Groups were matched by nonverbal developmental age and a factor for retention at follow‐up (FU; True vs. False) using nearest neighbor propensity score matching with a 1:4 ratio, allowing replacement and applying a caliper of 0.2. This ensured that differences in the longitudinal development are not confounded with systematic study dropouts between groups. Standardized mean difference and variance ratios pre‐ and post‐matching have been suggested as more accurate indicators of sample balance than mean difference testing (Ho et al. 2007; Zhang et al. 2019). Standardized mean differences below 0.1 and close to zero, and variance ratios below 2 and close to 1 indicate balanced samples (Zhang et al. 2019). Our matching procedure resulted in acceptable balance statistics (Table S1 in the Supporting Information). The final sample consisted of *n* = 115 children (*n*_ASD_ = 60; *n*_TD_ = 50). Sample descriptions of the unmatched samples can be found in Table S2. Informed consent was obtained by the parents and ethical approval was received by the local ethics committee (ethical vote for ASD: 19/18; TD: 361/18).

**TABLE 1:** Sample description of autistic and control group at baseline.

	Autism spectrum disorder (*n* = 60)	Typically developing controls (*n* = 55)
Data availability at FU	36 (60%)	29 (53%)
Sex (male/female)	49/11	25/30
Age (months)	47 (10); [26–64]	35 (14); [18–72]
Developmental age	29 (11); [16–66]	36 (15); [18–76]
Nonverbal IQ	62 (18); [31–108]	103 (11); [73–128]
SRS‐SF total	29 (7); [10–40]	5 (3); [0–12]
RBS‐R total	38 (26); [2–126]	9 (10); [0–54]
Months since baseline (FU)	38.4 (1.55)	30.4 (10.8)

*Note*: Mean (SD); [min–max].

### 2.2 Eye Tracking Apparatus and Procedure

Gaze behavior was evaluated using the Tobii TX300 eye‐tracker, which provides a gaze accuracy of 0.4 degrees of visual angle at a 300 Hz sampling rate. Eye‐tracking assessment was conducted under controlled and artificial lighting and at a screen distance of 50–80 cm. Participants were seated on a highchair or on their caregiver's lap in front of the presentation screen with a resolution of 1920 × 1080 pixels. They were instructed to focus on the screen. A five‐point calibration was performed. The study task took about 2 min to complete and was part of an eye‐tracking battery lasting approximately 25 min. The battery was programmed using Psychtoolbox‐3 for MATLAB (https://github.com/nicobast/BOSCA_battery).

### 2.3 Stimuli

**FIGURE 1:**
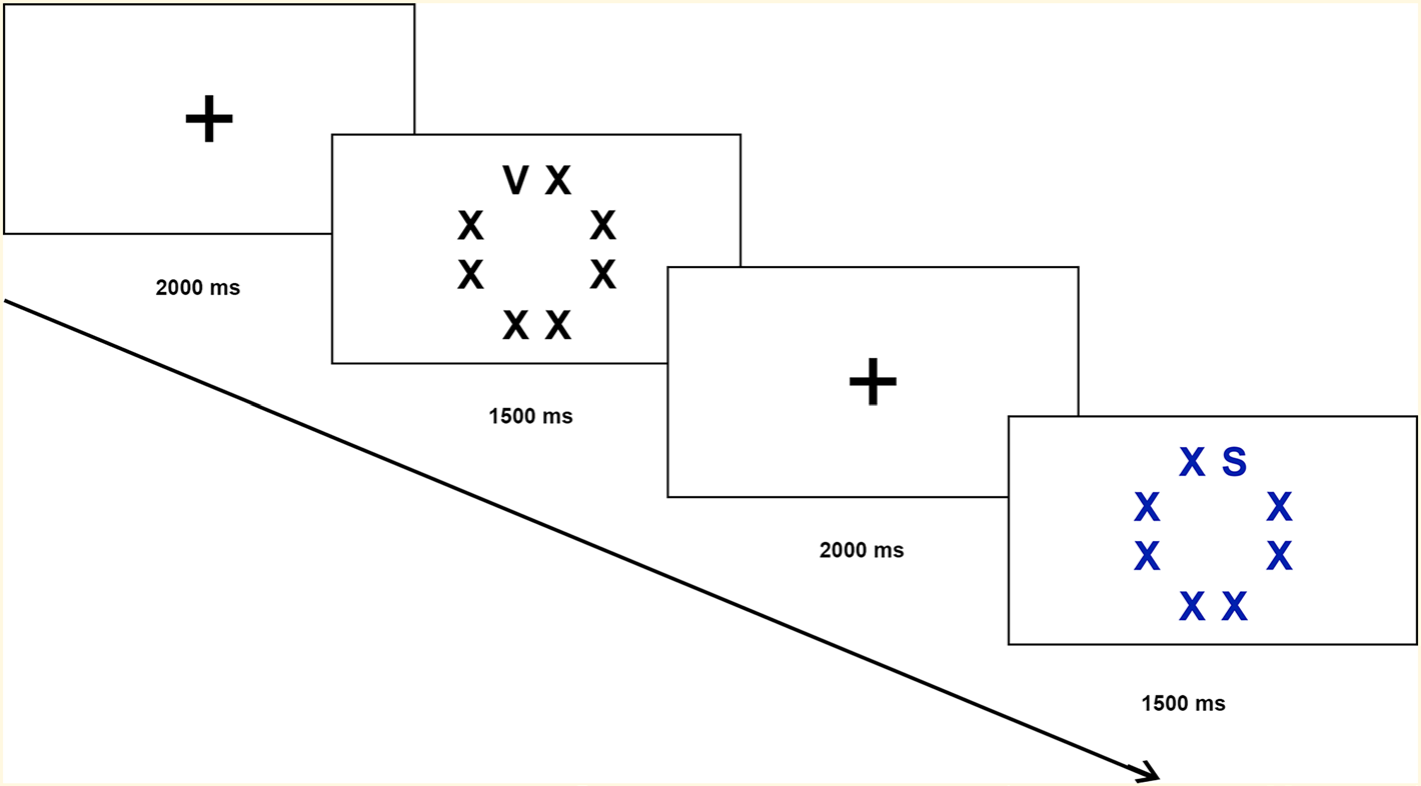
Stimulus material of the single feature visual search task. Illustration of the visual search task. Each trial consisted of a blinking fixation cross, followed by the stimuli. Total trial duration was 3 s. The color of stimuli and the position of the target alternated.

The stimulus material was based on Gliga et al. (2015) and consisted of arrays of eight letters situated on a circle on a white background (Figure 1). In each array, all but one stimulus were the same letter “X”. One stimulus, the target, was another letter (V, O, S, or H). The letters were 200 pixels in size, resulting in approximately 4.9 degrees of visual angle. The position of the target was varied pseudo‐randomly among the array (8 positions). Black, blue, or red sets of letters were alternated between trials to increase variability. We ensured that targets appeared equally at each location within the array. This uniform distribution was maintained to prevent any location bias and to ensure that each position was equally likely to contain a target. A total of 32 trials in blocks of 8 trials were shown, alternated with other tasks. Each trial had a duration of 3.5 s and consisted of the appearance of a blinking fixation cross in the screen‐center (2 s) followed by the stimuli (1.5 s).

### 2.4 Eye‐Tracking Data Processing

Raw eye‐tracking data were preprocessed following peer‐reviewed guidelines for gaze data (Nyström and Holmqvist 2010). Preprocessing algorithms are available online (https://github.com/nicobast/project_visualsearch/tree/main/code). Data were segmented by trial. Eye blinks lasting 75–250 ms and data points 25 ms before and after were excluded. Trials with less than 50% available data were excluded. Saccades were defined by a dynamic velocity‐based algorithm (i.e., high gaze position velocity indicates saccades) of the gaze position data depending on the data quality with an adaptive velocity cutoff range: 200–1000 degrees of visual angle per second. Within saccade detection, implausible velocity (*x* > 1000) and acceleration values (*x* > 100,000) were removed and velocity data was filtered with a Savitzky‐Golay filter of length 50 ms. Fixations were defined by gaze position differences less than 1 degree of visual angle over at least 100 ms including an absence of saccades.

Based on identified fixations, we calculated visual search outcome metrics. Accuracy (hit: True, False) was defined as a fixation on the target during a trial. The target area was defined by the target coordinates, including a tolerance of 15 pixels considering measurement variation. A fixation was considered a hit if it fell within the target area at least 100 ms after stimulus onset and had a minimum duration of 100 ms. Time to target was defined as the interval from stimulus onset to the first fixation on the target. Time to target values below 100 ms were considered coincidental and were excluded. Target fixation was based on the total fixation duration within the target area. Target fixation values below 100 ms were considered too short and were excluded. As a data quality measure, screen attention was computed based on all gaze positions on the presentation screen during a trial.

### 2.5 Behavioral/Other Measures

ASD core symptoms were assessed by the parent‐rated Social Responsiveness Scale Short Form (SRS‐SF; Sturm et al. 2017) and the Repetitive Behavior Scale‐Revised (RBS‐R; Lam and Aman 2007).

The SRS‐SF is a 16‐item short version of the full Social Responsiveness Scale (Constantino and Gruber 2012). Items are rated by the primary caregiver and scored on a scale from 0 to 3. Item scores are combined to a sum score, resulting in a score range of 0–48. Psychometric properties of the SRS‐SF are comparable to the full version (Lyall et al. 2022; Sturm et al. 2017).

The RBS‐R is a 43‐item questionnaire designed to capture a broad range of repetitive behaviors. Items are coded on a scale from 0 to 3. Here, we computed four subscales (insistence on sameness, repetitive sensory‐motor behavior, self‐injurious behavior, and compulsive behavior), based on a large trans‐diagnostic validation study (Kästel et al. 2021). In the SRS‐SF and the RBS‐R, higher scores indicate more pronounced symptoms.

To assess nonverbal cognitive ability, an adaptive procedure was applied since no single nonverbal cognitive measure spanning the age and developmental range is available in German. Children were assessed by the Bayley Scales of Infant Development‐III (Bayley‐III; Reuner and Rosenkranz 2014) or the Wechsler Preschool and Primary Scale of Intelligence‐III (Petermann et al. 2014) depending on age and instruction comprehension. Bayley‐III raw scores were directly transformed to developmental age. Nonverbal IQ was obtained by standardizing to chronological age and multiplying by 100. WPPSI‐III nonverbal cognitive raw scores were directly transformed to nonverbal IQ and developmental age.

### 2.6 Data Analysis

First, we assessed longitudinal group differences in visual search outcomes. Accuracy (hit likelihood) was investigated by generalized linear mixed models with a binomial link function. Time to target and target fixation were analyzed by linear mixed models. All models included group (ASD, TD), time point (baseline, FU), and their interaction as fixed effects. In all models, a random intercept for participant was included to account for inter‐individual differences and multiple trials. Prior to the analysis, age, sex, developmental age, and nonverbal IQ were ruled out as significant baseline covariates of accuracy and time to target in the complete sample (Tables S3 and S4). Thus, to keep the models comparable and parsimonious, only screen attention was further modeled as a fixed effect to account for data quality. In generalized linear mixed models, interaction effects between group and time point were examined by pairwise comparisons of estimated marginal means and 95% confidence intervals of odds‐ratio (OR). In linear mixed models, interaction effects were assessed by pairwise comparisons of estimated marginal means and 95% confidence intervals of standardized beta‐estimates (∆β), which can be interpreted as effect sizes.

Second, we assessed visual search performance as a predictor of ASD symptoms in the subsample of participants with an ASD diagnosis. We specified separate linear mixed models for the total scores of SRS‐SF and RBS‐R as dependent variables and visual search parameters (accuracy, time to target, target fixation) as fixed effects. Each model included fixed effects for the respective visual search parameter, time point, and their interaction. Nonverbal IQ was included as a covariate.

To rule out potential bias due to systematic dropouts between groups, dropout analyses were conducted by logistic regression (accuracy) and two‐way ANOVA models (time to target, target fixation) with predictors group (ASD, TD), retention status at FU (True, False), and their interaction.

## 3 Results

Descriptive statistics for accuracy, time to target, target fixation and screen attention at all time points are shown in Table 2. Screen attention per trial was sufficient at baseline (ASD: 70%; TD: 69%) and FU (ASD: 69%; TD: 74%).

**TABLE 2:** Descriptive statistics of visual search parameters at baseline and follow‐up.

		Autism spectrum disorder (*n* = 60)	Typically developing controls (*n* = 55)
Accuracy (hits)	Baseline	86% [33%–100%]	87% [43%–100%]
	FU	82% [33%–100%]	93% [50%–100%]
Time to target	Baseline	0.73 (0.36); [0.1–1.4]	0.74 (0.36); [0.1–1.38]
	FU	0.67 (0.34); [0.1–1.38]	0.83 (0.33); [0.1–1.4]
Screen attention	Baseline	2.78 (0.72); [0.14–3.49]	2.75 (0.78); [0.1–3.49]
	FU	2.69 (0.64); [0.46–3.46]	2.97 (0.62); [0.16–3.48]
Number of valid trials	Baseline	9 (4); [2–18]	10 (4); [2–19]
	FU	6 (3); [2–10]	8 (2); [2–11]

*Note*: Mean (SD); [min–max]. Values represent seconds if not otherwise specified.

### 3.1 Longitudinal Case‐Control Comparison

Main effects of screen attention were significant for accuracy and time to target. More attention on the screen is associated with higher accuracy (OR = 13.83 [5.57, 34.29], *p* < 0.01), and shorter time to target (*β* = −0.1 [−0.16, −0.03], *p* < 0.01). Full, unstandardized model estimates are shown in Tables S5 and S6.

**FIGURE 2:**
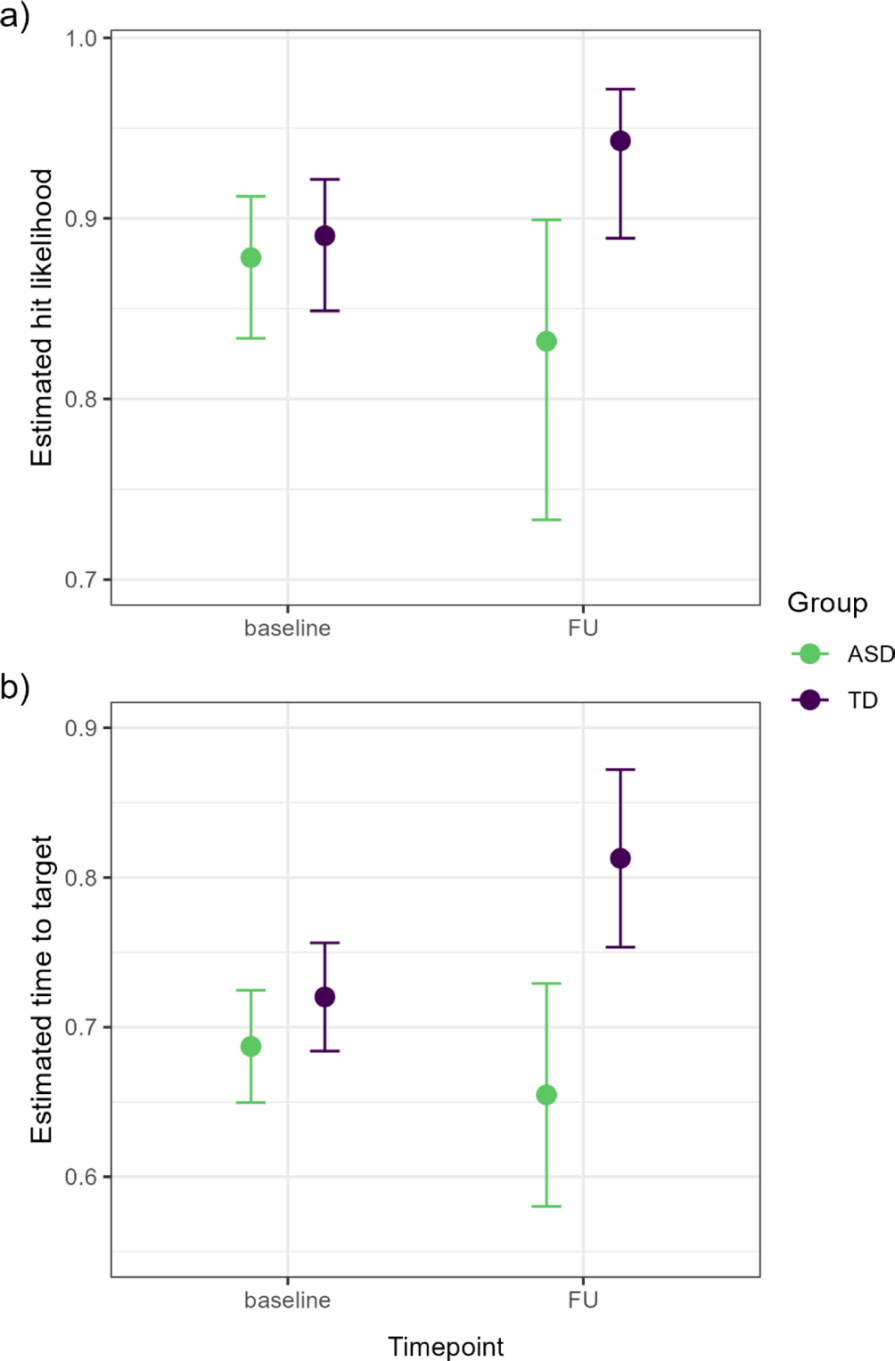
Marginalized mean of accuracy and time to target by group and time point, controlled for screen attention. Panel (a) shows the marginalized means of accuracy (likelihood of hit = True), predicted by a generalized linear mixed model with binomial link function, and panel (b) shows the marginalized means of time to target predicted by a linear mixed model.

For accuracy, we found a significant interaction effect between group and time point (OR = 2.97 [1.11, 7.94], *p* = 0.03). Accuracy was significantly lower in the ASD group than in the TD group at FU (OR = 0.30 [0.12, 0.76], *p* = 0.01) while there were no significant group differences at baseline (OR = 0.89 [0.54, 1.46], *p* = 0.64). At FU, the estimated marginal mean probabilities to correctly fixate the target were 83% [73%, 89%] in the ASD group and 94% [84%, 97%] in the TD group (Figure 2a). In the ASD group, accuracy decreased from baseline to FU (OR = 0.69 [0.36, 1.30], *p* = 0.25) while in the TD group accuracy increased from baseline to FU (OR = 2.03 [0.98, 4.24], *p* = 0.06), but neither change was statistically significant.

Regarding time to target, there was a significant interaction effect of group and timepoint (*β* = 0.35, SE = 0.15, *p* = 0.02). In post hoc comparisons, the ASD group showed significantly shorter time to target than the TD group at FU (contrast = −0.44, SE = 0.14, *p* < 0.01; Figure 2b) but not at baseline (*β* = −0.09, SE = 0.07, *p* = 0.21). Additionally, the TD group showed longer time to target at FU (*β*_FU_ = 0.08, SE = 0.03) compared to baseline (*β*_baseline_ = 0.73, SE = 0.02, *p*_contrast_ = 0.05).

### 3.2 Visual Search Performance and ASD Symptoms

**FIGURE 3:**
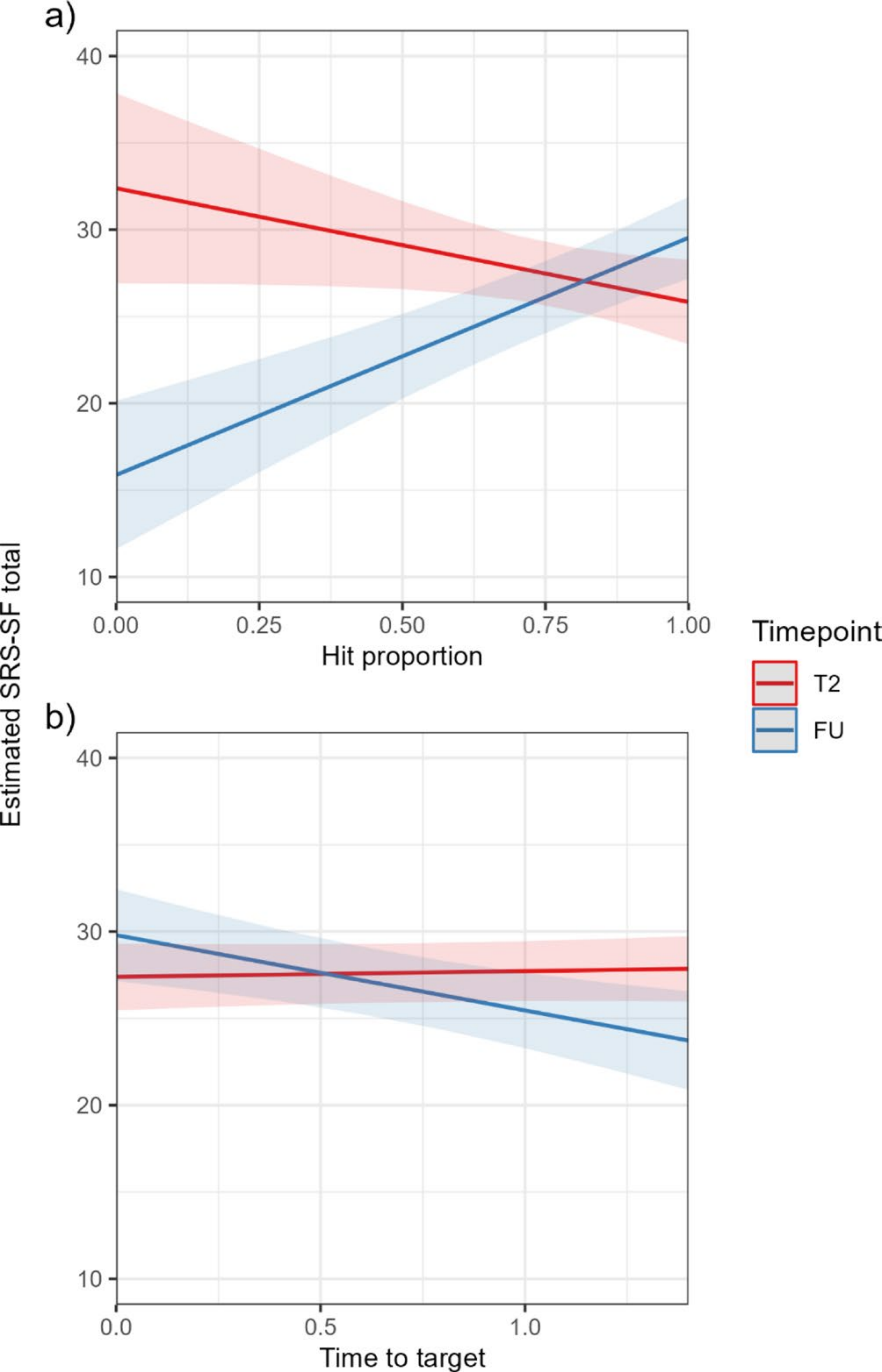
Preschoolers with autism spectrum disorder: SRS‐SF total score by visual search parameter and time point. The panels illustrate the interaction effect of each visual search parameter and timepoint on Social Responsiveness Scale‐Short Form (SRS‐SF) total score. Panel (a) shows the effect of accuracy (as proportion of hits over all trials) per timepoint. Panel (b) shows the effect of time to target in seconds. All values were predicted by linear mixed models with nonverbal IQ as an additional covariate.

For the SRS‐SF, we found a significant interaction effect between accuracy and time point (*β* = 0.63 [0.24, 1.02], *p* < 0.01), suggesting that the effect of accuracy on social communication varied by time point. At FU, higher accuracy was associated with higher SRS‐SF scores at FU (Figure 3a).

The interaction effect of time to target and time point on the SRS‐SF was also significant and shorter time to target indicated higher SRS‐SF scores (*β* = −0.24 [−0.40, −0.08], *p* < 0.01; Figure 3b). The full, unstandardized model estimates are shown in the Supporting Information (Tables S7 and S8).

RBS‐R total scores were significantly lower at FU (Table S9) but no significant main or interaction effects of visual search parameters were found. Post hoc power simulations (1000 iterations) of the linear mixed models for RBS‐R total score with the observed non‐significant effect size revealed that our sample might have been too small to detect significant interaction effects of time point with accuracy (Power = 15%), time to target (Power = 23%), and target fixation (Power = 66%).

To account for age variation in our sample, we re‐analyzed the data using age as a continuous predictor of ASD symptomatology instead of time point. Still, we found the same direction of effects and trends as with time point as a categorical predictor.

### 3.3 Dropout Analysis

No significant effects of group, retention status and their interaction were found for accuracy and time to target (Tables S10 and S11). In power analyses of the dropout models, the given sample size achieved a power of 59% (accuracy) and 84% (time to target, target fixation) to detect moderate effects (odds ratio = 1.75; *η* ^2^ = 0.07).

## 4 Discussion

In the present study, we examined longitudinal performance in a single‐feature visual search task over 3 years in preschoolers diagnosed with ASD in comparison to developmentally matched typically developing children. We evaluated accuracy and time to target as visual search performance measures. Additionally, we investigated the association of visual search accuracy and time to target with ASD symptom domains social communication and restricted and repetitive behavior over time.

High percentages of screen attention in both ASD and TD children indicated that participants were able to maintain similar levels of attention. Contrasting our expectations, our findings do not confirm the often‐reported advantage of ASD in visual search but show a more complex pattern. Both groups started from comparable levels of visual search accuracy and time to the target when our participants were in toddlerhood and preschool age. After 3 years, at elementary school age, participants with ASD showed less visual search accuracy but faster time to spot the target than TD children. These results have several implications. First, the identification of an ASD advantage or disadvantage in visual search appears to be dependent on the measure which is used to operationalize visual search performance. Meta‐analyses have described great heterogeneity in study designs and outcome measures which may have been the reason that effect sizes were small (Muth et al. 2014; Van der Hallen et al. 2015). Second, visual search performance may vary with age. Earlier studies reported age‐related improvements in visual search accuracy in school‐aged children with and without ASD using a cross‐sectional developmental design (Iarocci and Armstrong 2014). Also, studies which reported enhanced visual search in children with ASD in a similar age range to our sample were conducted with participants with average cognitive ability (Iarocci and Armstrong 2014; O'Riordan et al. 2001; Plaisted et al. 1998). Contrasting earlier studies, our study had a longitudinal design and predominantly included children with low IQ, which is representative for children diagnosed with ASD during preschool age (Denisova and Lin 2023). To account for low cognitive ability in the ASD sample, we chose to match our TD sample based on developmental age instead of chronological age as most previous studies did. Third, regarding the faster time to target in children with ASD, we might have observed an accuracy‐speed trade‐off. Typically developing children were more likely to spot the target, possibly at the cost of speed, while children with ASD were not as likely to spot the target, but if they were, they were faster to do so.

Interestingly, we found that higher visual search accuracy and faster time to target were associated with higher parent‐reported social communication issues. Visual search performance has been associated with enhanced perception and discrimination (Edmondson et al. 2020; Joseph et al. 2009). Our findings indicate that there may be no clear ASD advantage in these skills but within ASD, they may be associated with certain more severe symptom profiles. This may be a manifestation of the widely accepted broad heterogeneity within the autism spectrum.

In addition, in the ASD subsample, shorter time to target was significantly associated with higher social communication alterations at FU, which resonates with earlier findings of shorter reaction time in ASD (Jarrold et al. 2005; Joseph et al. 2009). We interpret this as an ASD advantage in visual search that may not be as pronounced as previously assumed. Especially in light of findings which described faster time to target to be a function of motor skills in children with ASD (Lindor et al. 2018), further research on possible correlates of enhanced visual search performance is needed to provide more insight into this often assumed “islet of ability” (Shah and Frith 1983).

Taken together, we found similar visual search performance in a single‐feature task and eye‐tracking assessment in preschool children with ASD diagnosis and TD children. Longitudinally, we showed that children with ASD showed lower visual search accuracy in elementary school age compared to their TD peers. However, children with ASD found the target faster. Within ASD, we found higher visual search accuracy and faster time to target to be related to more pronounced social communication aberrations.

Given the number of studies that have earlier reported the ASD advantage in visual tasks (e.g., Joseph et al. 2009; Kemner et al. 2008; O'Riordan et al. 2001), we interpret our findings as not contradicting earlier findings but we think that the ASD advantage in visual search may not be as pronounced as previously assumed. Especially in light of findings which described faster time to target to be a function of motor‐skills in children with ASD (Lindor et al. 2018), further research on possible correlates of enhanced visual search performance is needed to provide more insight into this often assumed “islet of ability” (Shah and Frith 1983).

### 4.1 Limitations

In our study, the data collection design was not completely parallel, since the interval between baseline and FU varied more among the TD group than in the ASD group (see Table 1). Also, not all participants could be followed up. Although our dropout analyses indicated that visual search accuracy and time to target values did not significantly vary as a function of group and retention status, dropout bias cannot be excluded. Regarding our stimulus material, we observed that both groups had high levels of accuracy. However, this may be related to the rather low number of valid trials as a result of the challenging nature of eye‐tracking assessments in younger populations. Variations of the experiment could have yielded more nuanced findings as previous studies have reported higher accuracy in a conjunct‐feature task but not in a single‐feature task (Kaldy et al. 2011). Finally, post hoc power simulation indicated that our sample was too small to detect significant effects of visual search parameters on restricted and repetitive behavior.

## Funding

This work was supported by Deutsche Forschungsgemeinschaft (FR 2069/8‐1, FR 2069/8‐2) and Goethe‐Universität Frankfurt am Main, (F38/21, R139/2021).

## Conflicts of Interest

C.M.F. receives royalties for books on ASD, ADHD and MDD. She has served as consultant for the German Institute for Quality and Efficiency in Health Care (Institut für Qualität und Wirtschaftlichkeit imGesundheitswesen, IQWiG) and the companies IGES and Infectopharm during the last 3 years. She has received research funding by the German Research Foundation (DFG), the German Ministry of Science and Education and the European Commission. N.B. receives royalties for lecturing at institutes for training in psychotherapy and receives third‐party funding by the German Research Foundation (DFG), the Daimler and Benz Foundation, and the Johanna Quandt Young Academy. The other authors declare no conflicts of interest.

## Supporting information


**Table S1:** Standardized mean difference and variance ratios pre and after matching of ASD and TD groups.
**Table S2:** Sample description of autistic and control group at baseline (unmatched sample).
**Table S3:** Odds ratios of fixating the target (accuracy) as predicted by sex, age, developmental age and nonverbal IQ by a generalized linear mixed model (unmatched sample).
**Table S4:** Estimated coefficients for time to target predicted by sex, age, developmental age and nonverbal IQ by linear mixed model (unmatched sample).
**Table S5:** Odds ratios of fixating the target (accuracy) as predicted by group, time point and their interaction using a generalized linear mixed model.
**Table S6:** Estimated coefficients for time to target predicted by linear mixed model.
**Table S7:** Estimated coefficients of SRS‐SF total score predicted by accuracy.
**Table S8:** Estimated coefficients of SRS‐SF total score predicted by time to target.
**Table S9:** Model estimates of the main effect of time point (FU) on RBS‐R total score.
**Table S10:** Model parameters of logistic regression of hit with predictors group, retention status, and their interaction.
**Table S11:** Two‐way ANOVA of time to target with predictors group, retention status and their interaction.

## Data Availability

The data that support the findings of this study are available from the corresponding author upon reasonable request.
