# Supplementary material for: Visual Search Performance in Children With ASD: A Combined Case‐Control and Longitudinal Study
Source: Autism Res. 2026 May 12;19(6):e70274. doi: 10.1002/aur.70274 (PMC13276684; doi:10.1002/aur.70274)
Supplement: Supplementary file 1 — Table S1: Standardized mean difference and variance ratios pre and after matching of ASD and TD groups. Table S2: Sample description of autistic and control group at baseline (unmatched sample). Table S3: Odds ratios of fixating the target (accuracy) as predicted by sex, age, developmental age and nonverbal IQ by a generalized linear mixed model (unmatched sample). Table S4: Estimated coefficients for time to target predicted by sex, age, developmental age and nonverbal IQ by linear mixed model (unmatched sample). Table S5: Odds ratios of fixating the target (accuracy) as predicted by group, time point and their interaction using a generalized linear mixed model. Table S6: Estimated coefficients for time to target predicted by linear mixed model. Table S7: Estimated coefficients of SRS‐SF total score predicted by accuracy. Table S8: Estimated coefficients of SRS‐SF total score predicted by time to target. Table S9: Model estimates of the main effect of time point (FU) on RBS‐R total score. Table S10: Model parameters of logistic regression of hit with predictors group, retention status, and their interaction. Table S11: Two‐way ANOVA of time to target with predictors group, retention status and their interaction. [file AUR-19-0-s001.docx]

# Supplementary material

# Visual search performance in children with ASD: a combined longitudinal and case-control study

## Table S1. Standardised mean difference and variance ratios pre and after matching of ASD and TD groups.

|  | pre-matching | | after matching | |
| --- | --- | --- | --- | --- |
|  | SMD | Variance ratio | SMD | Variance ratio |
| Developmental age | -0.77 | 0.48 | -0.16 | 0.74 |
| Retention status | -0.30 | - | 0.22 | - |

Note. SMD, standardised mean difference. Variance ratios are not stated for binary variables since their variance is a function of their mean.

## Table S2. Sample description of autistic and control group at baseline (unmatched sample)

|  | Autism Spectrum Disorder  (n = 60) | Typically developing controls  (n = 66) | *p* |
| --- | --- | --- | --- |
| Data availability at FU | 36 (60 %) | 30 (45 %) | <. |
| Sex (male/female) | 49/11 | 31/35 | <.001 |
| Age (months) | 47 (10); [26 - 64] | 36 (14); [18 - 77] | <.001 |
| Developmental age | 29 (11); [16 - 66] | 37 (16); [18 – 80] | <.001 |
| Nonverbal IQ | 62 (18); [31 - 108] | 103 (11); [73 - 128] | <.001 |
| SRS-SF total | 29 (7); [10 - 40] | 4 (3); [0 - 12] | <.001 |
| RBS-R total | 38 (26); [2 - 126] | 9 (10); [0 - 54] | <.001 |
| Months since baseline (FU) | 38.3 (1.52) | 30.1 (11.2) |  |

Note: Mean (SD); [min – max]. Wilcoxon Tests (age), chi square (sex)

## Table S3. Odds ratios of fixating the target (accuracy) as predicted by sex, age, developmental age and nonverbal IQ by a generalized linear mixed model (unmatched sample).

|  | Odds Ratio | *SE* | *p*-value |
| --- | --- | --- | --- |
| Intercept | 8.35 | 1.51 | **<.001** |
| Sex (female) | 0.71 | 0.19 | .20 |
| Developmental age | 0.86 | 0.45 | .77 |
| Age | 1.21 | 0.57 | .69 |
| Nonverbal IQ | 1.29 | 0.50 | .52 |
| **Variance components** |  |  |  |
| Residual variance | 3.29 |  |  |
| Random intercept | 0.53 |  |  |

n = 124, 963 observations

## Table S4. Estimated coefficients for time to target predicted by sex, age, developmental age and nonverbal IQ by linear mixed model (unmatched sample).

|  | Estimate | *SE* | *p*-value |
| --- | --- | --- | --- |
| Intercept | 0.78 | 0.17 | **<.001** |
| Sex (female) | 0.03 | 0.03 | .32 |
| Developmental age | 0.001 | 0.003 | .76 |
| Age | -0.001 | 0.003 | .75 |
| Nonverbal IQ | -0.0006 | 0.002 | .73 |
| **Variance components** |  |  |  |
| Residual variance | 0.15 |  |  |
| Random intercept | 0.003 |  |  |

n = 124, 963 observations

## Table S5. Odds ratios of fixating the target (accuracy) as predicted by group, time point and their interaction using a generalized linear mixed model.

|  | Odds Ratio | *SE* | *p*-value |
| --- | --- | --- | --- |
| Intercept | 1.16 | 0.40 | .68 |
| Group (TD) | 1.13 | 0.28 | .64 |
| Time point (FU) | 0.69 | 0.22 | .25 |
| Screen attention | 13.83 | 6.41 | **<.001** |
| Group (TD) x time point (FU) | 2.97 | 1.49 | **.03** |
| **Variance components** |  |  |  |
| Residual variance | 3.29 |  |  |
| Random intercept | 0.47 |  |  |

n = 115, 1115 observations

## Table S6. Estimated coefficients for time to target predicted by linear mixed model.

|  | Estimate | *SE* | *p*-value |
| --- | --- | --- | --- |
| Intercept | 0.84 | 0.05 | **<.001** |
| Group (TD) | 0.09 | 0.07 | .21 |
| Time point (FU) | -0.09 | 0.12 | .44 |
| Screen attention | -0.10 | 0.03 | **<.01** |
| Group (TD) x time point (FU) | 0.35 | 0.15 | .02 |
| **Variance components** |  |  |  |
| Residual variance | 0.98 |  |  |
| Random intercept | 0.002 |  |  |

n = 113, 966 observations

## Table S7. Estimated coefficients of SRS-SF total score predicted by accuracy.

|  | Estimate | *SE* | *p*-value |  |  |  |
| --- | --- | --- | --- | --- | --- | --- |
| Intercept | 36.28 | 1.61 | .001 |  |  |  |
| Nonverbal IQ | -0.13 | 0.02 | <.001 |  |  |  |
| Hit (True) | -0.46 | 0.65 | .48 |  |  |  |
| Time point (FU) | -4.01 | 1.25 | <.01 |  |  |  |
| Hit (True) x Time point (FU) | 4.33 | 1.37 | <.01 |  |  |  |
| **Variance components** |  |  |  |  |  |  |
| Residual variance | 16.02 |  |  |  |  |  |
| Random intercept | 38.52 |  |  |  |  |  |

n = 55, observations = 463.

## Table S8. Estimated coefficients of SRS-SF total score predicted by time to target.

|  | Estimate | *SE* | *p*-value |  |  |  |
| --- | --- | --- | --- | --- | --- | --- |
| Intercept | 35.22 | 1.59 | <.001 |  |  |  |
| Nonverbal IQ | -0.12 | 0.02 | <.001 |  |  |  |
| Time to target | 0.33 | 0.63 | .60 |  |  |  |
| Time point (FU) | 2.41 | 1.19 | .04 |  |  |  |
| Time to target x Time point (FU) | -4.67 | 1.54 | <.01 |  |  |  |
| **Variance components** |  |  |  |  |  |  |
| Residual variance | 16.11 |  |  |  |  |  |
| Random intercept | 37.32 |  |  |  |  |  |

n = 55, 463 observations

## Table S9. Model estimates of the main effect of time point (FU) on RBS-R total score.

| Model (VS parameter) | Estimate | *SE* | *p*-value |
| --- | --- | --- | --- |
| Accuracy | -5.13 | 1.21 | <.001 |
| Time to target | -3.32 | 1.15 | <.01 |

Note. VS, visual search. Models were separately specified with baseline RBS-R score, nonverbal IQ, the respective visual search parameter, time point and the interaction between visual search parameter and time point as fixed effects.

## Table S10. Model parameters of logistic regression of hit with predictors group, retention status, and their interaction.

| Predictors | Odds ratios | CI | *p* |
| --- | --- | --- | --- |
| Intercept | 5.74 | 4.94 – 8.40 | **<0.001** |
| Group | 0.90 | 0.55 – 1.45 | 0.66 |
| Retention status | 1.09 | 0.63 – 1.93 | 0.75 |
| Group x Retention status | 1.68 | 0.79 – 3.59 | 0.18 |

## Table S11. Two-way ANOVA of time to target with predictors group, retention status and their interaction.

|  | *df* | Sum Sq | Mean Sq | *F* | *p* |
| --- | --- | --- | --- | --- | --- |
| Group | 1 | 0.01 | 0.01 | 0.06 | 0.80 |
| Retention status | 1 | 0.15 | 0.15 | 1.14 | 0.29 |
| Group x Retention status | 1 | 0.01 | 0.01 | 0.10 | 0.75 |
| Residuals | 959 | 125.92 | 0.13 |  |  |
